# Tribulations and future opportunities for artificial intelligence in precision medicine

**DOI:** 10.1186/s12967-024-05067-0

**Published:** 2024-04-30

**Authors:** Claudio Carini, Attila A. Seyhan

**Affiliations:** 1 School of Cancer and Pharmaceutical Sciences, Faculty of Life Sciences and Medicine, New Hunt’s House, King’s College London, Guy’s Campus, London, UK; 2 Biomarkers Consortium, Foundation of the National Institute of Health, Bethesda, MD USA; 3 Laboratory of Translational Oncology and Experimental Cancer Therapeutics, Warren Alpert Medical School, Brown University, Providence, RI USA; 4 Department of Pathology and Laboratory Medicine, Warren Alpert Medical School, Brown University, Providence, RI USA; 5 Joint Program in Cancer Biology, Lifespan Health System and Brown University, Providence, RI USA; 6 Legorreta Cancer Center at Brown University, Providence, RI USA

**Keywords:** Artificial intelligence, Machine learning, Deep learning, Precision medicine, Drug discovery and development, Disease diagnosis, Prediction, Prevention, Health technology assessment

## Abstract

Upon a diagnosis, the clinical team faces two main questions: what treatment, and at what dose? Clinical trials' results provide the basis for guidance and support for official protocols that clinicians use to base their decisions. However, individuals do not consistently demonstrate the reported response from relevant clinical trials. The decision complexity increases with combination treatments where drugs administered together can interact with each other, which is often the case. Additionally, the individual's response to the treatment varies with the changes in their condition. In practice, the drug and the dose selection depend significantly on the medical protocol and the medical team's experience. As such, the results are inherently varied and often suboptimal. Big data and Artificial Intelligence (AI) approaches have emerged as excellent decision-making tools, but multiple challenges limit their application. AI is a rapidly evolving and dynamic field with the potential to revolutionize various aspects of human life. AI has become increasingly crucial in drug discovery and development. AI enhances decision-making across different disciplines, such as medicinal chemistry, molecular and cell biology, pharmacology, pathology, and clinical practice. In addition to these, AI contributes to patient population selection and stratification. The need for AI in healthcare is evident as it aids in enhancing data accuracy and ensuring the quality care necessary for effective patient treatment. AI is pivotal in improving success rates in clinical practice. The increasing significance of AI in drug discovery, development, and clinical trials is underscored by many scientific publications. Despite the numerous advantages of AI, such as enhancing and advancing Precision Medicine (PM) and remote patient monitoring, unlocking its full potential in healthcare requires addressing fundamental concerns. These concerns include data quality, the lack of well-annotated large datasets, data privacy and safety issues, biases in AI algorithms, legal and ethical challenges, and obstacles related to cost and implementation. Nevertheless, integrating AI in clinical medicine will improve diagnostic accuracy and treatment outcomes, contribute to more efficient healthcare delivery, reduce costs, and facilitate better patient experiences, making healthcare more sustainable. This article reviews AI applications in drug development and clinical practice, making healthcare more sustainable, and highlights concerns and limitations in applying AI.

## Introduction

The past 10 years have seen a remarkable acceptance of Artificial intelligence (AI) and Machine Learning (ML), which can help medical innovation for a more sustainable Precision Medicine (PM). The advantage of adopting AI and ML allows the analysis of extensive complex data, opening a new era for more sustainable healthcare. The potential of AI to generate insights from multi-dimensional data sets can support the use of PM in various diseases to discover new diagnostic and prognostic biomarkers.

AI works through ML, allowing computers to learn without being explicitly programmed for a specific task [1]. Indeed, if you feed the algorithm with enough good-quality data, ML will generate strategies for excelling at that task. However, so far, the power of AI to recognize sophisticated patterns and hidden structures has been limited to imaging and histopathology in the medical field.

With increasing costs, public pressure, and policy imperatives to manage patients across care episodes, the need to aggregate data across departments within and across different healthcare organizations is still an unmet medical need. The rapid explosion of AI has introduced the possibility of using aggregated healthcare data to produce robust models that can automate diagnosis [1] and also enable an increasing PM approach by tailoring treatments and targeting resources with maximum effectiveness on time (IBM and Partners to transform personal health with Watson and open cloud < https://www-03ibm.com/us/en/pressrelease/46580 was > 2015; [2]. The digitalization of healthcare data and the rapid uptake in technology are fueling transformation in the development and use of AI in healthcare [3].

However, "the truth" is that, at present, the algorithms that feature prominently in research are, in fact, not executable in clinical practice. This is happening for several reasons: 1. AI innovations do not re-engineer the incentives supporting existing working methods; 2 adding AI applications to an already fragmented healthcare system will not create sustainable healthcare changes; 3. most healthcare organizations lack the infrastructure required to collect the data to optimally train algorithms to (a) “fit” the local population and/or the local medical practice patterns, a requirement before the deployment rarely highlighted by current AI publications; (b) interrogate them for potential biases to guarantee that the algorithms perform consistently across patient cohorts, especially those who may not have been adequately represented in the training cohort [4]. An algorithm trained on mostly Caucasian patients is not expected to have the same accuracy when applied to minorities [5]. Rigorous evaluation and re-calibration must be done to capture those patient demographics that change over time [6]. Healthcare, with its abundance of data, is, in theory, well-poised to benefit from growth in cloud computing. The largest and most valuable store of data in healthcare rests in Electronic Medical Records (EMR). However, clinicians' satisfaction with EMRs remains low, resulting in variable completeness and data entry quality, and provider interoperability remains elusive [6]. The typical lament of clinicians is still, “Why is my EMR still inaccurate, and why don’t all these systems just talk to each other?”.

To value the potential of AI across health systems, more fundamental issues must be addressed: 1. who owns health data; 2. who is responsible for them; 3. who can use them? The potential of AI is well described in the literature [7]. However, in reality, health systems are faced with a choice to significantly downgrade the enthusiasm regarding the potential of AI in everyday clinical practice or to resolve data ownership and trust issues and invest in the data infrastructure to realize it.

AI and ML platforms have been extensively used in basic and clinical research spanning from drug discovery and development, diagnostic imaging, and genomic to other multi-omics data analysis, as reported recently by Liebman [8]. The utilization of AI technologies has become increasingly significant in accelerating various areas of biomedical research, including drug discovery and development, image-based disease diagnosis, and the analysis of large datasets, consequently enhancing decision-making processes across a wide range of fields and disciplines such as drug discovery, molecular biology, imaging, pathology, toxicology, and clinical medicine.

In particular, AI is on the rise in drug discovery and development. While advocates highlight the potential that these tools bring. Detractors, instead, adopt a more cautious approach, seeking solid evidence on the impact on drug discovery initiatives [9].

The right approach most likely lies right in the middle between those opposite views. Advances in the computational capability of AI have prompted concerns that AI technologies might replace physicians.

## AI in medicine

The real question is whether chatbots and large language model AI systems can reshape modern medicine or will lead to the opening of a Pandora's Box. Li et al. [10] consider different levels of healthcare applications for large language model AI systems, evaluating their capabilities and limitations, enable new workflows and models of care delivery, and shift the boundaries between human expertise vs artificial intelligence expertise. Li et al. [10] concluded that emerging AI systems could help to reduce the burden of laborious tasks in modern medicine, enabling physicians to devote their time to treating people.

Among the wide range of fields with possible applications of AI, medicine stands out as one with significant potential and substantial challenges. AI tools have become increasingly used in analyzing and interpreting large research databases ranging from laboratory findings to clinical data. All these tools offer the potential for increased efficiency and may unravel insights that are difficult to attain with traditional data-analysis methods. Despite a growing interest in deploying AI technologies in domains critical for sustainability, like healthcare, very few reports in the literature describe the potential systemic risks in depth [11, 12]. Unfortunately, using AI is not without strings, with social and ethical challenges to security, privacy, and human rights [13–15]. Disparities in disease care are due to the lack of affordable and inconsistent accessibility of patient data, especially in undeveloped countries. ML models are usually built on historical Caucasian data. Consequently, groups historically sidelined or experienced barriers to care can be affected by data, analytic and algorithmic bias. ML models in health care should be developed so that protected and non-protected demographic groups derive equal clinical benefits performing equally between the groups. During the evaluation phase of the algorithm, model performance should be assessed across different patient population groups. In addition, historical data on which the model is predicated should be assessed to determine whether these data would amplify and perpetuate racial bias. Unfortunately, only a handful of practical examples of AI medical use exist. However, the hype around this topic is unprecedented [7] with many AI papers published each year. Nevertheless, AI technology is still in its infancy in healthcare, and a short guiding medical professional to which clinicians can refer back is still lacking. Moreover, the proofs and evidence in favor of AI are yet to be convincing before AI gets adopted in medical practice extensively [7].

Undoubtedly, AI might benefit healthcare only when the medical community can assess its value and potential opportunities and acknowledge the limitations in treating different diseases [16]. Immunotherapy is now the standard treatment for cancer patients. However, many cancer patients do not respond to immune checkpoint inhibitors (ICI) treatment [17]. Predicting a response to ICI is still an unmet medical need. Identifying AI predictive biomarkers that can stratify ICI-treated patients in responders and non-responders is needed. AI biomarkers should be able to optimize patient stratification and minimize undesirable toxicities.

The power of AI technologies to recognize sophisticated patterns and hidden structures has enabled many image-based detection and diagnostic systems to perform as well as clinicians [18, 19].

However, whether AI enables clinical decisions and reduces diagnostic errors by assisting clinicians with EMR data extraction is yet to be proven [20, 21].

As highlighted in the recent AACR Cancer Progress Report 2022 [22], one area of intense research and rapid progress in recent years has been the use of AI and ML to analyze large amounts of imaging data collected for cancer screening. These technologies help recognize patterns that are often difficult to discern, even by trained experts. While further research is necessary, some AI-based medical devices and software systems have demonstrated high accuracy and effectiveness in clinical trials. For instance, between August 1, 2021, and July 31, 2022, the FDA approved several AI-enhanced software systems to assist clinicians in early cancer detection [22]. Table 1 illustrates a few examples of AI-based devices and software systems developed for detecting various types of cancers, including GI Genius for colorectal cancer, Paige Prostate for prostate cancer, Lunit INSIGHT MMG for breast cancer, and EndoScreener for colorectal cancer detection. For example, a breast cancer predicting algorithm, trained on 38,444 mammogram images from 9611 women, was the first to combine imaging and EMR data with associated health records. This algorithm could predict biopsy malignancy and differentiate between normal and abnormal screening results. The algorithm can be applied to assess breast cancer at a level comparable to radiologists, as well as having the potential to reduce missed diagnoses of breast cancer substantially [23]. Table 1 presents examples of several AI-enhanced software systems recently approved by the FDA to assist clinicians in early cancer detection [22].

**Table 1:** Examples of AI-based devices and software systems in cancer detection [22]

GI Genius	A medical device that uses AI-based software to assist clinicians in identifying precancerous lesions or polyps that may not be detectable during routine colonoscopy
Pelge Prostate	An AI-based software that reviews digitally scanned slide images from prostate biopsies to assist pathologists in the detection of areas that may be cancerous
Lunit INSIGHT MMG	An AI-based software that analyzes mammography images and provides the location of lesions suspected of being cancerous
Endoscreener	An AI-based software that identifies potentially precancerous polyps during a colonoscopy

Table 2 illustrates the examples of PubMed search results of published work in various cancer research fields that use AI in these publications. The words shown in the left column of the table have increased significantly since the first use of AI in any of the selected fields of medical research, including drug discovery, development, medicinal chemistry, cancer research, PM, etc. Data sources in the PubMed search included abstracts, original research articles, review articles, clinical trials, books and documents, and meta-analyses) (1960s-Oct. 22, 2023).

**Table 2:** Use of AI in various research fields from 1960s to 10-22-2023. Number of publications in various cancer research fields reported in PubMed ranging from 1960s to 10-22-2023. Data sources in the PubMed search included abstracts, original research articles, review articles, clinical trials, books and documents, meta-analyses

Disease fields	Number of publications (date range 1960s-Oct. 22, 2023)	Growth of AI use (1960s-Oct. 22, 2023)
Cancer research	17,395	
Healthcare	15,276	
Radiology	21,380	
	5347	
Drug discovery	4885	
Drug development	78,791	
Medicinal chemistry	2579	
Toxicology	1133	
Medical toxicology	373	
Drug design	3862	
Drug combination therapy	847	
Drug toxicity	1853	
Pharmacology	13,123	
Drug toxicity and safety	380	
Pharmacometrics	98	
Pharmacokinetics	3110	
Pharmacodynamics	13,202	
Biomarkers	10043	
Diagnostics	96,726	
Predictive	62,170	
Prognostic	4466	
Patient recruitment	2238	
Patient care	14,168	

Notably, there is exponential growth in the use of AI in all these fields. The first recorded use of automated pattern recognition goes back to a report published in the Lancet in 1960 [24].

Given the significance of AI in genomics and its potential impact on human health, a recent study [25] sought to evaluate factors that could improve the clinical application of AI in this field. The study has concluded that there is a significant need for informatics research and development to fully realize the clinical potential of these technologies. The creation of larger datasets is deemed essential to replicate the success seen by AI in other fields. It is imperative that AI techniques help to lessen rather than exacerbate the socioeconomic, racial, and ethnic divides that already exist. Establishing genomic data standards becomes imperative for the effective scalability of such technologies across institutions. Given the considerable uncertainty, complexity, and novelty in genomics and medicine, coupled with an evolving regulatory environment, the current emphasis should be on utilizing these technologies in collaboration with clinicians, highlighting the value each brings to clinical decision-making.

## AI in precision medicine

**Fig. 1:**
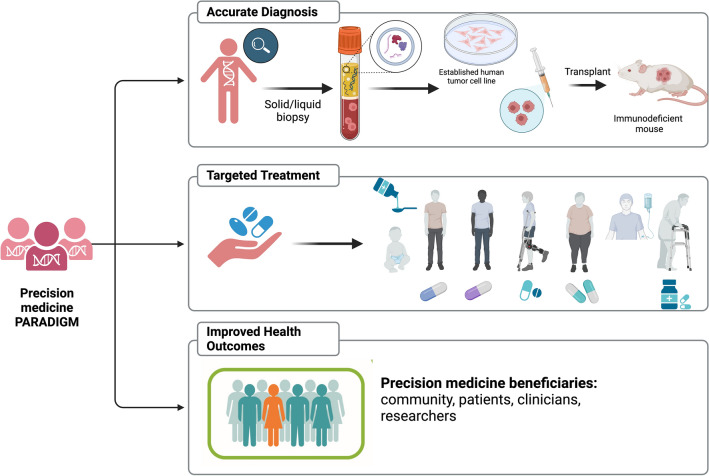
Precision medicine paradigm. Current approaches for precision medicine often involve assessment of various cancer drugs including chemotherapy, targeted therapies, or immunotherapy and others using patient derived tissue cancer cells or models such as spheroid or organoid as well as orthotopic murine xenograft models. With the rise of AI-based systems and technology platforms, it is anticipated that this process can be accelerated. Created with BioRender.com. (Accessed on 18 January 2024, 2024)

As reported in the literature [26–30] and illustrated in Fig. 1, one way to improve the value and efficiency of cancer and other disease therapy is by making PM an integral part of the approach to population health management. One-third of the EU adult population is currently affected by at least one chronic disease that contributes to 75% of mortality, and on average, 18 years of the last period of life are spent with at least one disability [31].

Approaches to PM are already being implemented for diseases like cancer, both in the diagnosis and treatment. Regrettably, we are still in the infancy of preventing and predicting diseases in healthy individuals. Several PM applications can have the potential for more effective prevention of chronic diseases, postponing the onset of disabilities and reducing healthcare costs [32]. Over the last twenty years, the incredible progress in genotyping technology, the reduction in genome sequencing costs, and the advent of digital technologies in healthcare, including wearable devices to monitor health, have initiated a third revolution in medicine. In this context, there is an increasing interest in finding informative markers that indicate the disease risk before the symptomatic manifestations of the disease occur (primary prevention) or for early disease detection (secondary prevention).

Clinicians have used genotype information as a guideline to help determine the correct dose of warfarin [33]. The Clinical Pharmacogenetics Implementation Consortium published genotype-based drug guidelines to help clinicians optimize drug therapies with genetic test results. [34]. Genomic profiling of tumors can inform targeted therapy plans for patients with breast or lung cancer [35]. PM and AI integrated into healthcare have the potential to yield more precise diagnoses, predict disease risk before symptoms occur, and design customized treatment plans that maximize safety and efficiency.

As previously reported by Johnson, 2021 [5], the trend toward enabling the use of PM by establishing data repositories is not restricted to the United States; examples from Biobanks in many countries, such as the UK Biobank, [36], BioBank Japan, [37], and Australian Genomics Health Alliance [38] demonstrate the power of changing attitudes toward PM globally. It is known that there is a certain synergy between AI and PM. They both impact the goal of personalizing care in several ways: therapy planning using clinical, genomic, or social and behavioral determinants of health and risk prediction/diagnosis using genomic or other variables.

Although there is much promise for AI and PM, more work still needs to be done to test, validate, and change treatment practices. Researchers face challenges in adopting unified data formats (e.g., Fast Healthcare Interoperability Resources), obtaining sufficient and high-quality labeled data for training algorithms, and addressing regulatory, privacy, and socio-cultural requirements.

## AI in drug discovery and development

**Fig. 2:**
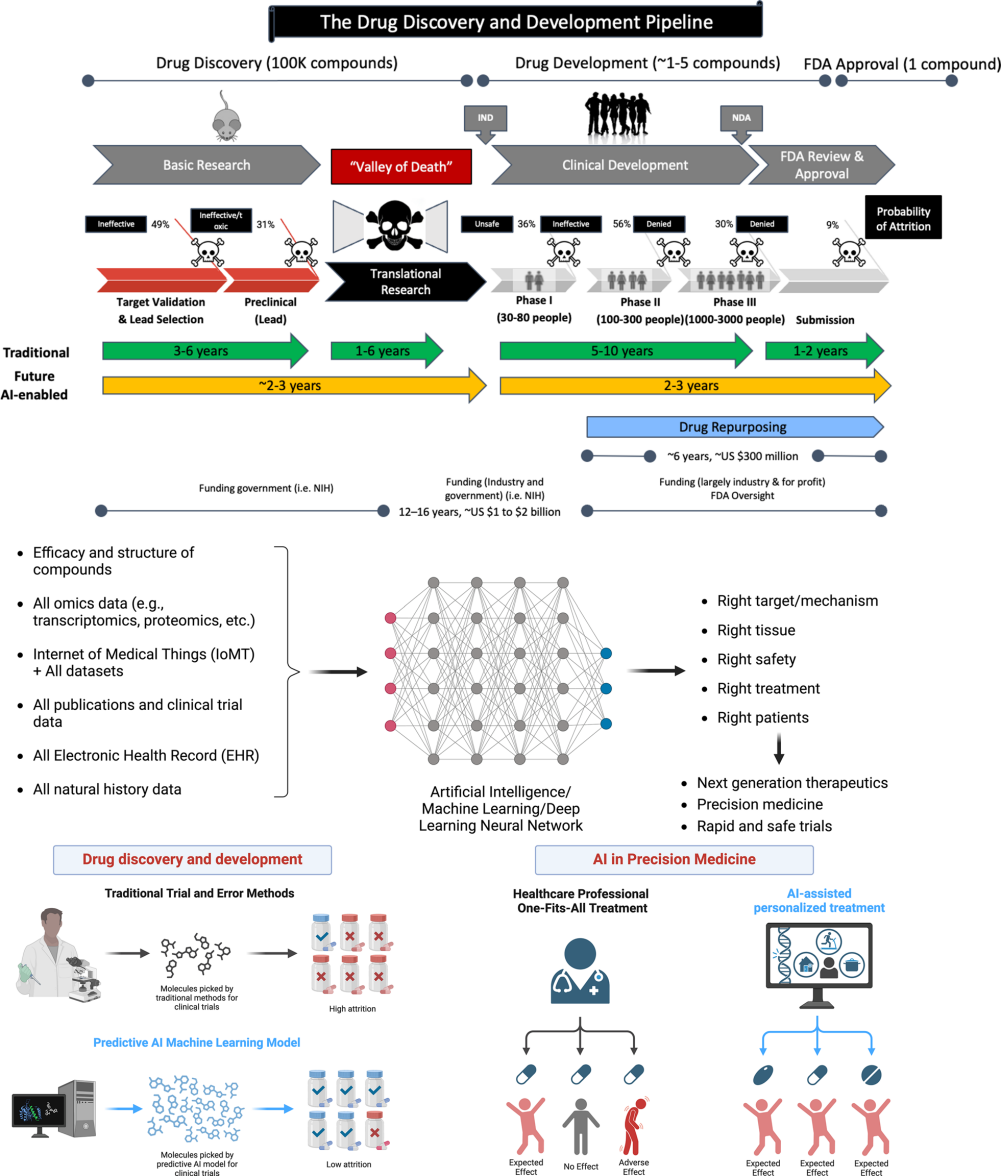
AI-based technologies can accelerate the drug discovery and development process and reduce the cost. Left panel: AI-based techniques can accelerate the drug discovery and development process, potentially reducing the attrition rate, time, and the cost. AI-based drug discovery and screening alongside laboratory automation could augment human drug design, chemical synthesis, drug screening, biological testing, and decision-making in design–make–test–analyze cycles involved in drug discovery and development, potentially overcoming low success rates, long drug development process, and high-cost often associated with traditional drug discovery and development process. Right panel: in clinical trial space, AI-based techniques can help physicians to leverage patient’s genomic data to identify suitable drugs that target those genomic aberrations. This approach offers the potential for enhanced drug effectiveness, improved safety profiles, decreased adverse reactions, expanded treatment choices, and, ultimately, a potential for saving lives. Abbreviations**: **DNN, deep neural network; EHR, electronic health records; IoMT, internet of medical things; ML, machine learning. Created with BioRender.com. (Accessed on 18 January 2024, 2024).

**Fig. 3:**
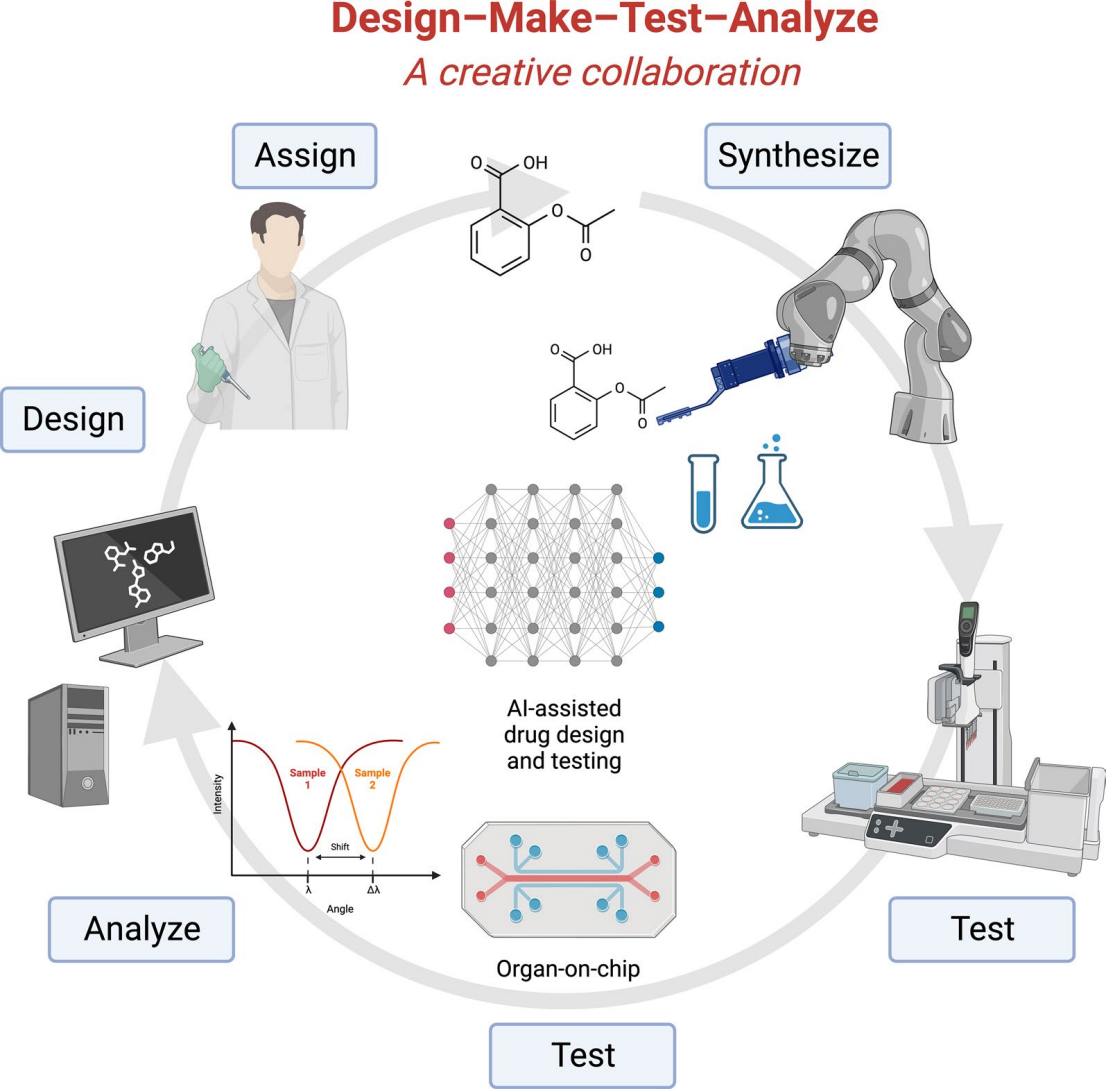
AI in drug discovery and laboratory automation for preclinical testing. AI-based drug discovery and screening alongside laboratory automation could augment human drug design, chemical synthesis, drug screening, biological testing, and decision-making in design–make–test–analyze cycles involved in drug discovery. Created with BioRender.com. (Accessed on 18 January 2024, 2024)

As illustrated in Figs. 2 and 3 and reported in the literature [22, 26–29, 39], the timeline from drug target and discovery to phase 1 human clinical trials and, ultimately, FDA approval, followed by Phase 4 studies indeed can go on for several years. Given that the costs and time necessary to develop a drug have become unsustainable, there is an urgent need to accelerate drug discovery and development and reduce the cost and time it takes to register a drug.

In the upcoming years, the adoption and application of AI techniques (machine learning, deep neural networks, and multifaceted biomedical AI) is expected to accelerate clinical research significantly. AI will affect drug discovery transformation, enhancing image interpretation, streamlining electronic health records, optimizing workflow, and gradually progressing the field of public health.

As discussed in the literature, [27, 28, 39–42], AI-based drug discovery and screening alongside laboratory automation could augment human drug design, chemical synthesis, drug screening, biological testing, and decision-making involved in drug discovery and development, potentially overcoming low success rates, long drug development process, and high costs often associated with traditional drug discovery and development process (Fig. 2).

As highlighted in recent literature [42], with the advancement of therapeutic strategies [43], the field of drug discovery and development is adopting innovative methodologies like data science, informatics, and AI, among others. These developments aim to improve efficiency, lower costs, and reduce reliance on animal testing, thus accelerating the creation of new and potent therapies. The intersection of big data and AI in drug discovery continues to attract considerable interest [44]. Investors [45, 46], industry experts [47, 48], researchers [49, 50], and policymakers [51] are actively participating in discussions on the implications of AI for drug discovery.

The successful approval of a drug requires the concurrent optimization of various properties encompassing pharmacokinetics (PK), pharmacodynamics (PD), and clinical outcomes. PK entails absorption, distribution, metabolism, excretion, and toxicity (ADMET), while PD aspects pertain to drug-target interactions and efficacy along with drug safety considerations. Clinical outcomes encompass therapeutic intentions, as delineated by the list of drug indications and off-label uses, as well as undesired effects such as side effects or adverse drug reactions.

Therefore, the successful campaign of a drug discovery program relies on three fundamental pillars: diseases, targets, and therapeutic modalities and AI influences most of these therapeutic modalities, such as antibodies [52], gene therapy [53], oligonucleotide [54], targeted protein degradation [55], and vaccine [56] design.

In human clinical trial space, AI-based methods can help physicians leverage patients' genomic data to identify drugs targeting those genomic aberrations. This approach offers enhanced drug effectiveness, improved safety profiles, decreased adverse reactions, expanded treatment choices, and a potential for saving lives (Fig. 2).

As discussed in the literature [9] and illustrated in Fig. 3 illustrates how integrating artificial intelligence and laboratory automation can enhance human decision-making and improve the processes of chemical synthesis and biological testing processes within the design-make-test-analyze cycles integral to drug discovery. This collaborative intelligence, resulting from the synergy of human expertise and machine capabilities, is expected to lead to more informed decision-making.

The use of AI enabled drug discovery and development, clinical trial design and enrollment through drug discovery, interpreting imaging, streamlining electronic health records, and improving workflow, advancing public health over time. AI can help in many of these aspects at all stages of the drug development process, including the different phases of human clinical trials.

## AI in clinical trials

As recently reported by Kumar et al. [57], many clinical trial studies have adopted AI to improve cancer screening/diagnosis and predict treatment outcomes. These studies rely on digital pathology, radiology, and genomic data to optimize the design of combination regimens and determine appropriate dosing of chemotherapy and immunotherapy [58–60]. As highlighted in the literature [5], there are several challenges concerning AI systems that could affect the successful transition to real-world healthcare. These challenges include fairness and bias, socio-environmental factors, data safety, and privacy. Additionally, Table 3 presents various pros and cons of AI systems in precision medicine, emphasizing other critical points to consider.

**Table 3:** Pros and cons of AI-based technologies in PM

Pros		Cons	
Improved diagnostics	AI can analyze vast amounts of medical data, including patient records, imaging results, and genetic information, to assist in detecting and classifying diseases with greater accuracy and speed	Lack of large, well-annotated cancer datasets	The Lack of large, well-annotated cancer datasets that represent a diverse patient population, distinct cancer burdens across various subset of populations is a main barrier for the effective use of AI in cancer research and patient care (Ref: AACR Cancer Progress Report, 2022, p. 53)
More accurate cancer diagnosis	Combining conventional blood tests and liquid biopsies with AI-assisted analyses can lead to more accurate cancer diagnosis	Data privacy and security	The use of AI in medicine requires collecting and storing vast amounts of sensitive patient data, raising concerns about data privacy and security breaches
Personalized treatment	AI can help create personalized treatment plans for patients based on their individual medical history, genetic makeup, lifestyle, and other factors, leading to more effective and tailored interventions	Lack of human touch	While AI can improve efficiency, some patients may prefer human interactions and emotional support, which AI systems cannot fully provide
Predictive analytics	AI Algorithms can forecast disease trends and patient outcomes, enabling healthcare providers to anticipate and prevent potential health issues before they escalate	Bias in algorithms	AI algorithms heavily rely on the data they are trained on, which may contain inherent biases. If not adequately addressed, these biases could lead to unequal treatment and perpetuate existing healthcare disparities
Enhancing drug development	AI can aid in drug discovery by identifying potential drug candidates, predicting their effectiveness, and expediting the research and development process	Overreliance on technology	Dependence on AI systems might lead to complacency among healthcare professionals, reducing their ability to make critical decisions independently
Remote patient monitoring	AI-powered devices can enable remote monitoring of patients, allowing healthcare providers to track health metrics and intervene promptly if any concerning trends are identified	Legal and ethical challenges	The introduction of AI in medicine raises complex legal and ethical questions concerning liability, accountability, and the ethical use of patient data
Equity and equality	AI-powered devices can improve equity and equality or worsen it–see cons	Cost and implementation challenges	Integrating AI into healthcare systems can be expensive, and smaller healthcare facilities might face challenges adopting and maintaining AI-driven technologies

As of October 18, 2023, there were (1584 clinical trials and 910 observational and 655 interventional) across various diseases reported on Clincaltrials.gov that used AI (Table 4). In the neurologic disorders, there were a total of 170 studies (94 of them observational and 76 interventional) across various neurologic disorders, including Alzheimer's disease, Parkinson's, CNS tumor, stroke, diabetic neuropathies, disease, and many others reported in Clincaltrials.gov that use AI. Rheumatic Diseases: Only 6 clinical trials (2 observational and 4 interventional) across Rheumatic Diseases were reported in Clincaltrials.gov that use AI. Of which, studies. Cardiovascular Diseases: There were 276 clinical trials (187 observational and 89 interventional) in cardiovascular diseases (also searched for disorders, diagnoses, and conditions) reported on Clincaltrials.gov that used AI.

**Table 4:** As of October 18, 2023, there were only five Phase 1, 15 Phase 2, and only three Phase 3 clinical studies globally that use AI

Row	Status	Study Title	Conditions	Interventions	Study Type	Phase	NCT Number
1	Not yet recruiting	Optimizing and Personalising Azacitidine Combination Therapy for Treating Solid tumors QPOP and CURATE.AI	● Solid Tumor ● Gastrointestinal Cancer ● Breast Cancer	● Device: QPOP ● Device: CURATE.AI ● Drug: Azacitidine + docetaxel ● (and 2 more…)	Interventional	Phase 1 Phase 2	NCT05381038
2	Active, not recruiting	Evaluating the Performance of AI in Evaluating Breast MRI Performed With Dose Reduction	● Breast Benign Tumor ● Breast Malignant Tumor	● Drug: Standard of Care (SOC) gadolinium Breast MRI ● Drug: reduced 1/4 dose gadolinium Breast MRI with Artificial Intelligence (AI) to aid in evaluation	Interventional	Phase 1	NCT04340180
3	Recruiting	PRECISE CURATE.AI Pilot Clinical Trial	● Solid Tumor	● Device: CURATE.AI ● Drug: Capecitabine ● Drug: XELOX ● (and 2 more…)	Interventional	Phase 1 Phase 2	NCT04522284
4	Recruiting	Nivolumab and Pembrolizumab Dose Optimisation in Solid Tumors With CURATE.AI Platform and Sequential ctDNA Measurements	● Solid Tumor	● Device: CURATE.AI ● Drug: Nivolumab, Pembrolizumab	Interventional	Phase 1 Phase 2	NCT05175235
5	Not yet recruiting	AD HOC Trial: Artificial Intelligence-Based Drug Dosing In Hepatocellular Carcinoma	● Hepatocellular Carcinoma	● Drug: Irinotecan ● Drug: Sonidegib ● Drug: Sorafenib	Interventional	Phase 1	NCT05669339
6	Recruiting	Artificial Intelligence Supporting Cancer Patients Across Europe—the ASCAPE Project	● Breast Cancer ● Prostate Cancer ● Quality of Life ● (and 2 more…)	● Other: ASCAPE-based follow-up strategy	Interventional	Phase 2	NCT04879563
7	Recruiting	Hyperpolarized 13C Pyruvate MRI for Early Immune Evaluation in Cervical Cancer Patients at Baseline and CCRT Therapy	● Cervical Cancer	● Drug: Hyperpolarized 13C Pyruvate	Interventional	Phase 2	NCT04951921
8	Unknown	Neoadjuvant Camrelizumab, Nab-paclitaxel and Carboplatin in Stage IB-IIIA NSCLC	● Lung Cancer, Non-small Cell ● Artificial Intelligence	● Drug: Camrelizumab + Nab-paclitaxel + Carboplatin	Interventional	Phase 2	NCT04541251
9	Not yet recruiting	Optimizing and Personalising Azacitidine Combination Therapy for Treating Solid Tumors QPOP and CURATE.AI	● Solid Tumor ● Gastrointestinal Cancer ● Breast Cancer	● Device: QPOP ● Device: CURATE.AI ● Drug: Azacitidine + docetaxel ● (and 2 more…)	Interventional	Phase 1 Phase 2	NCT05381038
10	Not yet recruiting	This Study is Evaluating a New Radiation Treatment Technique for Patients Who Have Had Prostate Cancer, Undergone Surgery for Cancer, and Then Have Evidence That Their Prostate Cancer Has Returned	● Recurrent Prostate Cancer After Surgery	● Radiation: Daily-adaptive Stereotactic Body Radiation Therapy	Interventional	Phase 2	NCT05946824
11	Recruiting	PRECISE CURATE.AI Pilot Clinical Trial	● Solid Tumor	● Device: CURATE.AI ● Drug: Capecitabine ● Drug: XELOX ● (and 2 more…)	Interventional	Phase 1 Phase 2	NCT04522284
12	Completed	Imatinib Mesylate in Treating Patients With Refractory or Relapsed Ovarian Epithelial, Fallopian Tube, or Primary Peritoneal Cancer, or Ovarian Low Malignant Potential Tumor	● Fallopian Tube Cancer ● Ovarian Cancer ● Primary Peritoneal Cavity Cancer	● Drug: imatinib mesylate	Interventional	Phase 2	NCT00039585
13	Not yet recruiting	Hyperpolarized 13C MRI for Cancer Immunotherapy	● Gynecologic Cancer ● Cervical Cancer ● Endometrial Cancer ● Ovarian Cancer	● Drug: Hyperpolarized 13C-Pyruvate injection	Interventional	Phase 2	NCT05805358
14	Not yet recruiting	PSMA PET Scan and mpMRI for Prostate Cancer Detection	● Prostate Cancer Diagnosis	● Diagnostic Test: PSMA PET scan ● Other: No PSMA PET ● Drug: 18F- DCFPyl Injection	Interventional	Phase 2	NCT05820724
15	Not yet recruiting	Compare the Efficacy and Safety of Intranasal Esketamine in Chronic Opioid Refractory Pain	● Cancer Pain	● Drug: esketamine nasal spray ● Drug: placebo nasal spray	Interventional	Phase 2	NCT04666623
16	Recruiting	Nivolumab and Pembrolizumab Dose Optimisation in Solid tumors With CURATE.AI Platform and Sequential ctDNA Measurements	● Solid Tumor	● Device: CURATE.AI ● Drug: Nivolumab, Pembrolizumab	Interventional	Phase 1 Phase 2	NCT05175235
17	Completed	Gefitinib in Treating Patients With Cervical Cancer	● Cervical Cancer ● Fallopian Tube Cancer ● Ovarian Cancer ● Primary Peritoneal Cavity Cancer	● Drug: gefitinib ● Other: immunohistochemistry staining method ● Other: surface-enhanced laser desorption/ionization-time of flight mass spectrometry ● (and 2 more…)	Interventional	Phase 2	NCT00049556
18	Terminated	Basket Trial in Solid Tumors Harboring a Fusion of FGFR1, FGFR2 or FGFR3- (FUZE Clinical Trial)	● Solid Tumor	● Drug: Debio 1347	Interventional	Phase 2	NCT03834220
19	Active, not recruiting	INRT-AIR: A Prospective Phase II Study of Involved Nodal Radiation Therapy	● Head and Neck Squamous Cell Carcinoma	● Radiation: Intensity modulated radiation therapy (IMRT)	Interventional	Phase 2	NCT03953976
20	Not yet recruiting NEW	DCIS: RECAST Trial Ductal Carcinoma In Situ: Re-Evaluating Conditions for Active Surveillance Suitability as Treatment	● Ductal Carcinoma in Situ	● Drug: Tamoxifen ● Drug: Exemestane ● Drug: Letrozole ● (and 3 more…)	Interventional	**Phase 2**	NCT06075953
21	Recruiting	Total Neoadjuvant Therapy of SCRT + CAPOX vs SCRT + CAPOXIRI for Locally Advanced Rectal Cancer (ENSEMBLE)	● Locally Advanced Rectal Cancer	● Radiation: SCRT ● Drug: CAPOX ● Drug: CAPOXIRI	Interventional	Phase 3	NCT05646511
22	Recruiting	Two Studies for Patients With High Risk Prostate Cancer Testing Less Intense Treatment for Patients With a Low Gene Risk Score and Testing a More Intense Treatment for Patients With a High Gene Risk Score, The PREDICT-RT Trial	● Metastatic Malignant Neoplasm in the Bone ● Prostate Adenocarcinoma ● Stage III Prostate Cancer AJCC v8 ● (and 4 more…)	● Drug: Apalutamide ● Drug: Bicalutamide ● Drug: Buserelin ● (and 9 more…)	Interventional	Phase 3	NCT04513717
23	Recruiting	A Study to Compare Standard Therapy to Treat Hodgkin Lymphoma to the Use of Two Drugs, Brentuximab Vedotin and Nivolumab	● Lugano Classification Limited Stage Hodgkin Lymphoma AJCC v8	● Procedure: Biospecimen Collection ● Biological: Bleomycin Sulfate ● Drug: Brentuximab Vedotin ● (and 17 more…)	Interventional	Phase 3	NCT05675410

In the oncology field, there were 452 clinical trials (271 observational and 181 interventional) reported in Clincaltrials.gov that use AI. Notably, the documented applications of AI commonly involve the oncology field and are primarily used in recruitment [61]. As the trial outcomes continue to be realized if AI will ultimately change practice in oncology, several factors that extend far beyond technology and data will need to be explored.

As of October 18, 2023, only five studies were found for AI in cancer in Phase 1 and 15 studies were found in Phase 2, and only 3 AI studies were found for cancer in Phase 3. On the other hand, no studies were found for AI in Phase 4 cancer trials. Notably, most of these studies noted above involved diagnostic tests, devices, and others not included in the Phase 1–3 studies.

## AI in cancer diagnosis

The utilization of AI in various fields has experienced exponential growth, with the first recorded automated use of pattern recognition dating back to a report published in the Lancet in 1960 [24]. In the context of cancer diagnosis, current literature [22, 62, 63] reveals numerous studies exploring AI's potential by comparing their results to manual detection by pathologists. AI demonstrates a notable degree of accuracy, surpassing human pathologists in diagnosing specific types of cancer [64–67]. AI effectively detected precancerous colonic polyps, leading to a two-fold reduction in missed identifications compared to pathologist diagnoses when using traditional colonoscopy [62]. The FDA's recent approval of AI for cancer early detection and diagnosis underlines the efficacy of the AI approach. AI applications' AI extends to predicting the likelihood of developing metastasis, as demonstrated in a study on bone metastasis in breast cancer patients, where an AI algorithm correctly predicted bone metastasis likelihood in 88% of cases [68]. Ensuring accurate and equitable AI-based screening requires broad application across diverse groups, including racial and ethnic minorities. A meta-analysis of AI programs detecting melanoma revealed a lack of disclosure regarding skin type and race/ethnicity in many studies. Without inclusive data on darker skin colors and reporting of race and ethnicity, AI algorithms can lead to biased technologies with inconclusive or false diagnoses. Efforts must be made to reduce biases in technologies by incorporating a health equity lens early in development, increasing recruitment and representation of diverse populations in AI clinical trials, and implementing reporting standards and auditing [69–72]. Inclusive AI algorithms, such as Mirai which utilize data from global populations, demonstrate high accuracy in predicting breast cancer development across diverse countries [72]. Reducing biases in AI technologies is crucial for maximizing their effectiveness and ensuring health equity.

A recent study [73] has reported findings from an AI system trained to conduct medical interviews, demonstrated performance equal to or exceeding that of human doctors in conversing with simulated patients and suggesting possible diagnoses based on patients’ medical history. The chatbot, developed on Google's large language model (LLM), exhibited greater accuracy than board-certified primary-care physicians, particularly in diagnosing cardiovascular and respiratory conditions. During medical interviews, the AI system gathered a comparable volume of information to human doctors and demonstrated higher levels of empathy.

## Barriers to the use of AI in cancer diagnosis

As previously reported by Johnson et al. [5] here, we describe some of the main challenges involving AI systems that would impact the success of the transition to real‐world healthcare: “fairness and bias, socio‐environmental factors, data safety and privacy” as well as other points focusing of pros and cons of AI systems in PM highlighted in Table 3.

These statements highlight essential challenges and considerations in the intersection of AI, healthcare, and biomedical research. Let's delve into some key points:

## 1. Bias in health data and AI models

- Challenges: Biases in health data, such as underrepresentation or missing values, can lead to biased AI models. This bias can result in unfair and unfavorable decisions for specific demographic groups.
- Solutions: Initiatives like the All of Us program, focusing on diverse participant recruitment, aim to enhance data diversity. The AI community is actively researching techniques to detect and address bias in models (source).
- Call to Action: Further exploration and collaboration between the AI and biomedical communities are essential to understanding and mitigating bias in AI models trained on historical patient data.

## 2. Data safety and privacy

Challenges: Data safety and privacy are critical considerations for an AI-driven system. As AI and PM intersect, collecting and integrating diverse data types, including genomics, medical history, behaviors, and social data covering individuals' daily lives, will become more prevalent.

Because of this, privacy concerns among individuals are closely linked to trust in using AI-enabled services.

Solution: Establishing a secure and well-regulated ecosystem for data storage, management, and sharing is imperative.

Call to Action: Adopting new technologies, collaborations, and developing innovative regulations and business models.

## 3. Domain-specific considerations

- Challenges: Fairness and protected aspects are closely tied to the specific domain and applications. Biomedical research requires a nuanced examination of fairness and bias in AI models.
- Solutions: Tailoring solutions to the context of biomedical research, acknowledging the unique challenges and considerations within the domain.

## 4. Social and environmental factors

- Challenges: environmental factors and deployment workflow can influence AI model performance. Prospective studies, such as the one on diabetic retinopathy screening, highlight the impact of real-world conditions on AI system effectiveness.
- Issues faced: Diverse clinic conditions, internet connectivity issues, and travel-related concerns can affect AI model performance and user participation.
- Solutions: Validation of AI models in real-world clinical settings, iterative feedback loops, and system enhancements based on user feedback are crucial for successful deployment. Examples reported by groups like Baowaly et al. [74] demonstrate considerable promise, but additional AI research efforts are warranted.

## 5. Iterative model validation and user feedback

- Importance: The example of the AI system for diabetic retinopathy screening emphasizes the significance of ongoing validation in real-world scenarios and the incorporation of user feedback.
- Recommendation: Establishing iterative loops that gather user feedback can inform continuous learning and improvement of AI systems before widespread application.

In summary, addressing bias and considering real-world conditions are crucial for ethically and effectively deploying AI models in healthcare. Collaborative efforts, ongoing validation, and context-specific solutions ensure fairness and optimal performance in biomedical AI applications.

## AI in cancer treatment

**Fig. 4:**
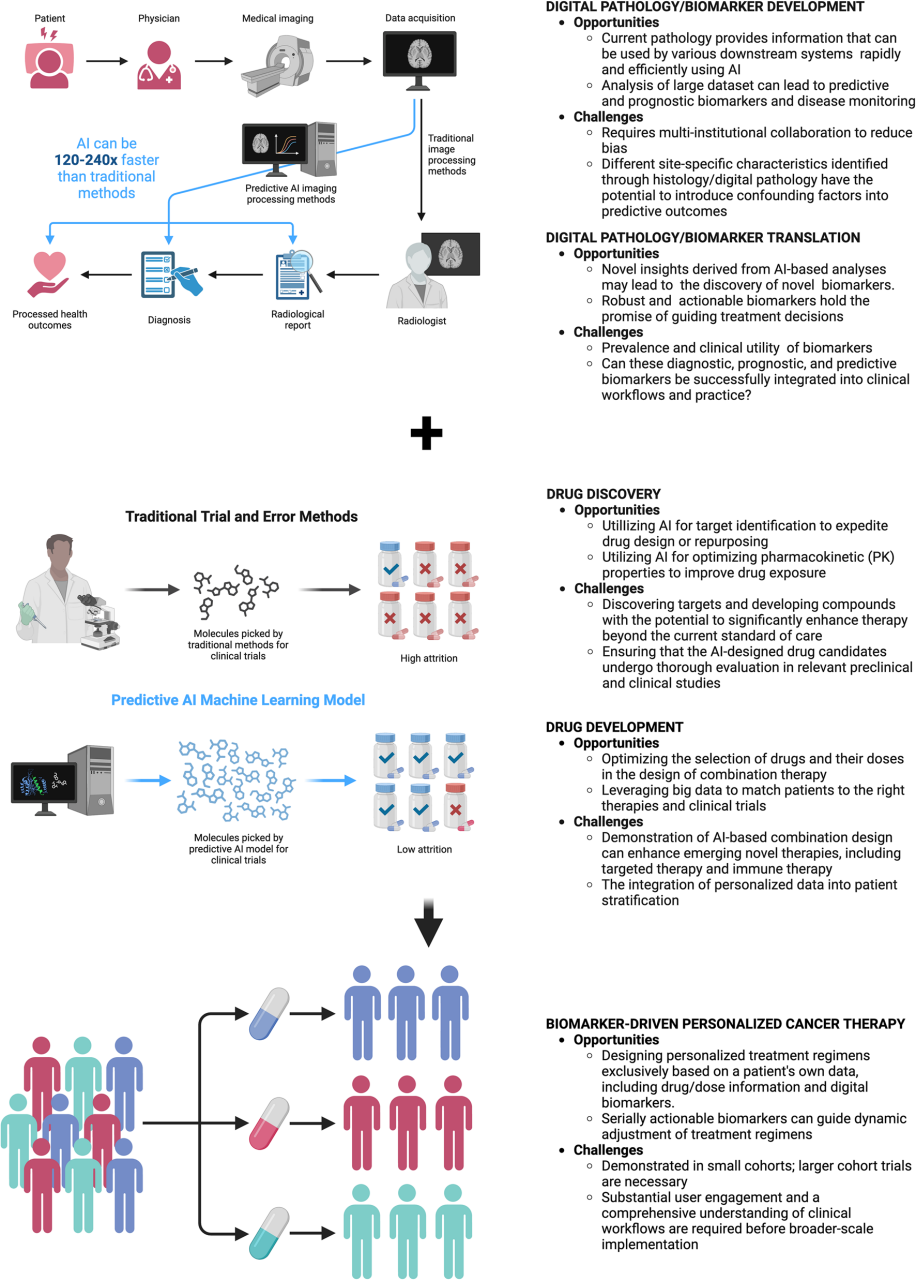
Leveraging AI for personalized treatment. AI-focused workflow explores the opportunities and challenges of applying AI in digital pathology, drug discovery and development, and dynamic drug dosing. Created with BioRender.com. (Accessed on 18 January 2024, 2024)

**Fig. 5:**
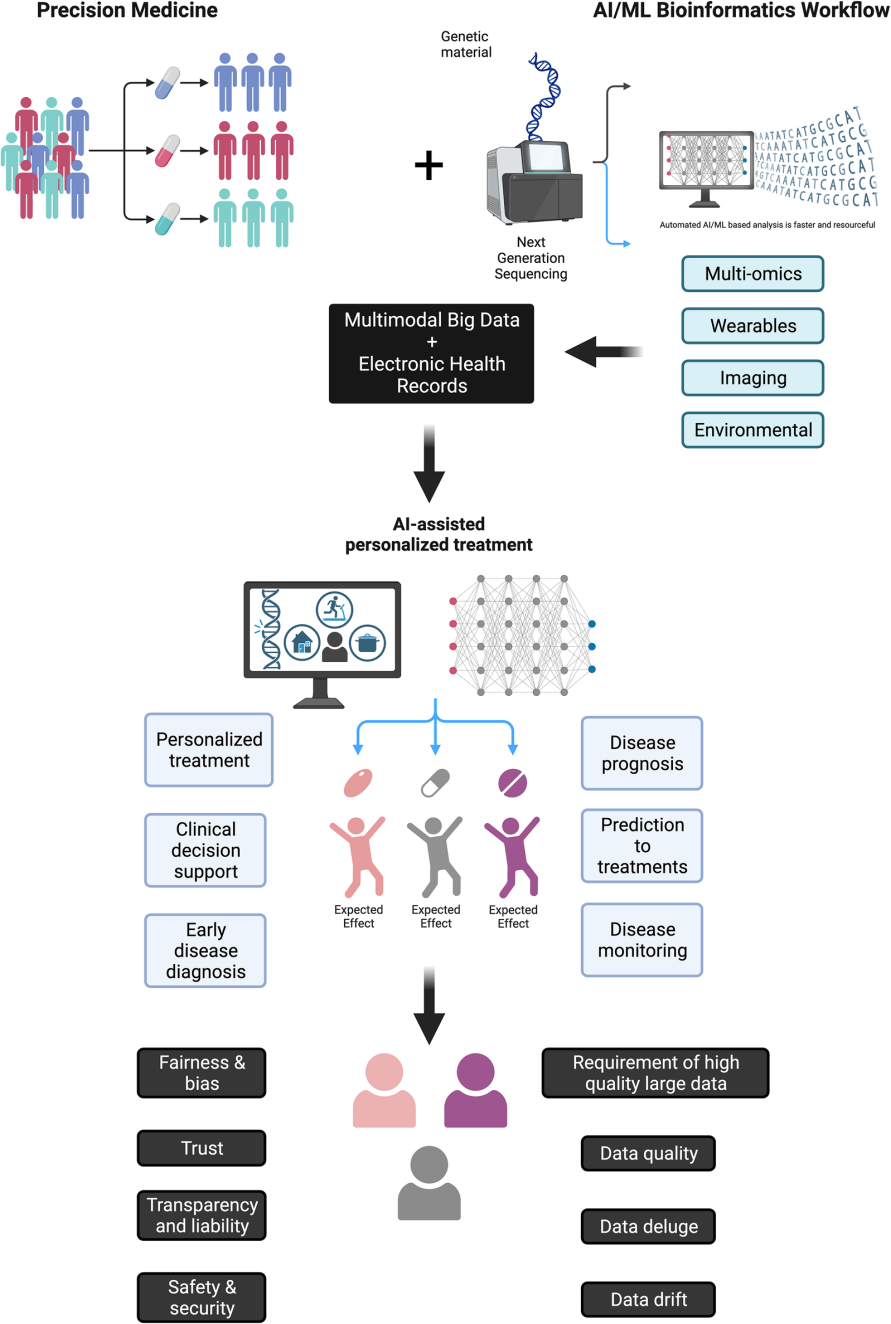
Illustration of integration of AI into PM. Recent attempts to integrate AI into PM have demonstrated significant potential and progress in personalized care, clinical decision support systems, early disease detection, and disease monitoring. However, there are outstanding challenges and concerns involving technical challenges and ethical issues and concerns (e.g., fairness and bias, trust, transparency and liability, trust, safety and security, as well as requirement of high quality large data, data deluge, and data drift) that may hinder the progress and reliability of the field and delay clinical implementation. Created with BioRender.com. (Accessed on 18 January 2024, 2024)

As discussed in the literature [22, 57] and illustrated in Fig. 4, the prevailing PM approach takes into account a range of factors, including tumor-associated and inherited genetic variations, environmental exposures, lifestyle, general health, and medical history of patients, when determining the most suitable treatment plans for individual patients [75]. On the contrary, as AI gains traction in PM, we anticipate its expanding role across various critical areas, from diagnosis to drug discovery and development, and in matching patients with targeted drugs tailored to their specific genomic or genetic alterations (Figs. 4 and 5). Recent efforts to incorporate AI into PM have shown substantial promise and advancements in personalized care, clinical decision support systems, early disease detection, and disease monitoring. However, persistent challenges and issues related to technology and ethics (such as fairness and bias, transparency and liability, trust, safety, and security as well as requirement of high quality large data, data deluge, and data drift) could impede the field's progress and reliability, potentially delaying clinical implementation (Fig. 5) [5, 76].

For example, in a study utilizing AI to create a radiotherapy regimen for prostate cancer, 89% of the radiotherapy treatment plans generated for the 100 patients studied were deemed clinically acceptable. Impressively, 72% of these plans were superior to those devised by human experts [77]. Another study applied AI to identify patients with head and neck cancers who would benefit from reducing the intensity of radiotherapy or chemotherapy. AI accurately predicted which patients would benefit from treatment de-escalation [78].

## Examples of successful drug discovery efforts facilitated by AI

Recent literature [42, 79] highlights the demonstrated potential of AI in the three pillars of drug discovery: diseases, targets, and therapeutic modalities, as evidenced by various studies.

Traditional drug discovery methods often depend on identifying and modifying existing compounds, a process that is slow, laborious, and costly. In contrast, AI-based approaches have the potential to facilitate the rapid, efficient, and cost-effective discovery and design of novel compounds with desirable PK, PD, and ADME properties and activities. For instance, a DL algorithm was used to train a dataset containing known drug compounds and their associated properties. This resulted in the identification of new therapeutic molecules with desirable traits such as solubility and activity, demonstrating the utility of these AI-based methods for rapidly, efficiently, and cost-effectively designing new drug candidates [80].

AlphaFold, a powerful algorithm that uses protein sequence data and AI to predict the proteins’ corresponding three-dimensional structures [81] uses protein sequence data and AI to predict the proteins’ corresponding three-dimensional structures which potentially can revolutionize personalized medicine and drug discovery by providing unprecedented insights into protein structures.

For example, a recent study has demonstrated [67] the effectiveness of AI in uncovering novel cancer treatment compounds. They trained a DL algorithm on a large dataset of known cancer-related compounds and their biological activity. This approach yielded promising compounds with potential for future cancer therapies, highlighting the method's ability to identify new therapeutic options. Additionally, the use of machine learning to pinpoint small-molecule inhibitors of MEK, a potential cancer treatment target, has been documented [82]. Similarly, AI has been employed to identify inhibitors of beta-secretase (BACE1), a protein associated with Alzheimer's disease [83].

Moreover, AI has facilitated the discovery of new antibiotics, with a pioneering machine learning approach identifying potent antibiotic types from a pool of over 100 million molecules, including compounds effective against tuberculosis and resistant bacterial strains [84, 85].

In the realm of COVID-19 research, AI has emerged as a promising tool. For example, ML algorithms have been used to analyze large datasets of potential compounds to identify those with the greatest potential for treating the virus.

Notably, these AI-powered approaches have significantly reduced the time needed to identify promising drug candidates compared to traditional methods [86–91]. Numerous other examples highlight the capacity of AI-based methods to expedite drug screening and discovery [92] and enhance the development of more effective therapies, drug combinations for drug synergies [93, 94], and drug repurposing [88, 95–103] for various other diseases [104–110].

Furthermore, ML plays a significant role in predicting drug efficacy and toxicity. As highlighted in recent literature [79], a DL algorithm was recently trained using a dataset of known drug compounds alongside their corresponding biological activity [111]. Subsequently, the algorithm demonstrated high accuracy in predicting the activity of novel compounds. Significant progress has also been made in preventing the toxicity of potential drug compounds through intensive training using extensive databases of known toxic and non-toxic compounds for using ML [112].

Another significant application of AI in drug discovery involves identifying drug-drug interactions that occur when multiple drugs are combined for the same or different diseases in a single patient, potentially leading to altered effects or adverse reactions. This issue has recently been tackled by an ML algorithm, which accurately predicts the interactions of novel drug pairs [113].

As highlighted in recent literature by groups like Hasselgren and Oprea [42] ChatGPT, a conversational AI that has successfully passed the US Medical Licensing Examination, to revolutionize research practices and publishing [114]. The GPT-4 technical report from March 14, 2023, demonstrates its capability to create new drugs, among other applications [115]. In an experiment, GPT-4 was prompted with the drug name dasatinib and tasked with modifying it, identifying similar compounds, locating vendors, and arranging custom synthesis if necessary.

Notable observations include GPT-4's ability to generate valid chemical structures (Simplified Molecular Input Line Entry System, SMILES) output [116], demonstrating GPT-4’s capability to accurately perceive and modify chemical structures. Furthermore, it successfully identifies molecules availability in the ZINC database [117], indicating their synthetically feasible nature. While the proposed molecule, desmethyl-imatinib, was not novel, GPT-4 successfully modified the molecule while retaining its kinase inhibitor properties.

However, experimental validation is required to confirm whether the GPT-4-generated molecule shares the same mode of action as dasatinib. Although GPT-4 has general expertise and is not specifically tailored for drug discovery, tools like ChemCrow [118], a GPT-4-based tool demonstrate how external resources can enhance Large Language Models (LLM) effectiveness in chemistry-related tasks. Integration of more external resources with GPT-4 or its successors could further enhance their capabilities in chemistry-related domains.

## Examples of big pharma engaging in AI-driven drug discovery, development, and clinical trial efforts

As highlighted in recent literature [79], the utilization of AI algorithms to analyze data from large populations enables AI researchers and pharmaceutical scientists to uncover crucial trends and patterns. These insights aid in predicting the efficacy of potential drug candidates for specific patient populations, thus tailoring treatments to individual needs.

For example, the collaboration between Merck and Numerate, an AI company, exemplifies this approach, with a focus on developing AI-based strategies for medicinal chemistry [119]. In this rapidly growing research domain, numerous new companies are emerging, poised to make significant short-term impacts [120].

A recent study highlights successful partnerships between AI firms and the pharmaceutical industry in drug discovery and development [121]. For example, 11 major pharmaceutical companies are leveraging AI platforms to transform drug discovery, optimize clinical trials, identify novel drug targets, generate lead compounds, and enhance manufacturing processes [120]. Sanofi has partnered with Aily Labs and a French startup AI company, Hillo. Pfizer has teamed up with IBM, and Novartis has partnered with Microsoft and NVIDIA. Janssen has introduced its Trials360.ai service. AstraZeneca has partnered with Oncoshot, and Bristol Myers Squibb and Bayer have both joined forces with Exscientia. Merck has expanded its partnerships to include BenchSci, Atomwise, C4 Therapeutics, and ACMED, while GSK has partnered with Cloud Pharmaceuticals and Insilico Medicine. Roche has partnered with Recursion Pharmaceuticals, and Lilly has recently signed an AI partnership with Alphabet's Isomorphic [122].

Such partnerships highlight the pharmaceutical industry's commitment to utilizing AI platforms to enhance efficiency, cut costs, and ultimately advance patient outcomes.

Through these collaborations, these entities can identify novel targets for drug development and improve the efficacy of existing treatments, thereby impacting clinical practice, and benefiting patients while enhancing their quality of life.

## AI in drug-matchmaking

An AI-based UK-based company, Exscientia, is testing a new patient-drug matchmaking technology that pairs individual patients with the precise drugs they need, considering the subtle biological differences between people [123].

The researchers used a small sample of tissue from a patient, dividing the sample, which included both normal cells and cancer cells, into more than a hundred pieces and exposing them to various cocktails of drugs. Using robotic automation and machine-learning models trained to identify small changes in cells led to an exhaustive search for the right drug and identified a runner-up in the matchmaking process: a cancer drug marketed by the pharma giant Johnson & Johnson that was not effective at treating this type of cancer in previous cancer trials. This drug worked in a patient with a specific pattern, and the patient was in complete remission for the last two years. In addition to matching patients up with existing approved or experimental drugs, the company uses machine learning to design new drugs, which could provide even more options when looking for a match. The first drugs designed with the help of AI are now in clinical trials to see if a treatment is safe and efficacious. Exscientia isn’t alone. There are now hundreds of startups exploring the use of machine learning in the pharmaceutical industry.

On average, it takes more than 10 years and billions of dollars to develop a new drug. The AI-assisted approach can make drug discovery and testing faster and cheaper by predicting how potential drugs might behave in the body and selecting those with potential while eliminating those compounds that may fail before they leave the design stage. Machine-learning models can reduce the need for complex, long, and costly lab work. And there is always a need for new drugs.

## AI leads to suspicions and optimism

As highlighted recently in Helio.com by Volansky R. [124] and in a recent paper by Li et al., [10], there are several issues regarding using AI to analyze large batches of complicated data. One of the risks is rapidly propagating false and biased information from these sources. In other words, AI is expected to interpret flawed data, generate inadequate results, and ultimately affect a treatment strategy in the clinic. However, if inaccurate data are used in research, the results could cause a flawed conclusion about the efficacy and safety of a compound. Significant progress in health care has been made since 2009 with the adoption of the electronic medical record (EMR) to recent advances in AI and ML [124]. But as AI and machine learning start to make their interpretation of data, the risk of medical malpractice will increase. A recent report [125] noted that the most significant benefits of AI methods are seen with unstructured data frequently found in rheumatology, such as images and text, where traditional ML systems were not as effective in analyzing large amounts of information held within these data formats. In another recent study presented at the American College of Allergy, Asthma & Immunology Annual Scientific Meeting, ChatGPT answered accurately or somewhat accurately 91% of the time when asked about 10 allergy myths [126]. In the same study, in a survey gauging the potential utility of ChatGPT, allergists rated 70% of its responses to questions regarding allergy myths as accurate and 21% as precise somewhat [126]. Additionally, nearly half of the allergists intended to utilize chatbots for patient education [126].

Addressing the challenges posed by individual variations in treatment response and the complexities of combination therapies in clinical practice requires a multifaceted approach that integrates advances in AI, personalized medicine, and clinical research. Several strategies must be considered to address those challenges. Implement robust data quality assurance processes to ensure the accuracy, completeness, and reliability of healthcare datasets that train and validate AI algorithms. This includes data cleaning, normalization, and validation procedures to identify and correct errors, inconsistencies, and missing values that may compromise the integrity of the data. Several parameters should be required when adopting AI: 1 Data quality and anonymization, encryption, and differential privacy are required to safeguard sensitive patient information and mitigate the risk of unauthorized access or data breaches; 2 Developing and implementing strategies to mitigate biases in AI algorithms and ensure fair and equitable treatment across diverse patient populations. This includes conducting thorough algorithmic audits, evaluating model performance across different demographic groups, and addressing training data biases through data augmentation, bias detection, and algorithmic fairness testing; 3 Adhere to regulatory requirements and industry standards for the responsible development, validation, and deployment of AI technologies in healthcare; 4 Establish ethical guidelines and governance frameworks to guide AI's responsible and ethical use in healthcare, including principles of beneficence, non-maleficence, autonomy, and justice; 5 Engage stakeholders from diverse backgrounds, including patients, clinicians, ethicists, and policymakers, in developing ethical guidelines and decision-making frameworks that prioritize patient welfare and uphold ethical standards; 6 Implement continuous monitoring, evaluation, and feedback mechanisms to assess the performance, safety, and efficacy of AI-driven healthcare interventions in real-world clinical settings. 7 Monitor key performance indicators, adverse events, and patient outcomes to identify potential risks, gaps, and areas for improvement in AI-enabled healthcare delivery.

## AI and sustainable healthcare

Overcoming obstacles related to cost, implementation, and data annotation is crucial for maximizing AI's benefits in clinical medicine. Here are some tools and strategies that can be employed to overcome obstacles**:** 1 Open-source tools and libraries; 2 Cloud computing platforms; 3 Transfer learning; 4 Collaborative annotation tools; 5 Clinical data registries; 6 Collaboration and partnerships. By leveraging these tools and strategies, healthcare organizations can address obstacles related to cost, implementation, and data annotation, thereby unlocking the full potential of AI in clinical medicine.

Controlling costs while maximizing the benefits of AI in medicine requires a combination of strategic approaches. Here, we listed some strategies that can be implemented to optimize AI benefits: 1 Identify and prioritize AI applications in medicine that can potentially deliver significant benefits in terms of patient outcomes and cost savings. Targeting areas, such as medical imaging interpretation and predictive analytics for disease diagnosis and treatment planning, can yield substantial returns on investment; 2 Collaborate with healthcare institutions, AI developers, researchers, and regulatory bodies to share resources, expertise, and best practices; 3 Invest in robust data management infrastructure and interoperability standards to effectively collect, store, and integrate healthcare data from disparate sources; 4 Implement mechanisms for continuous evaluation and improvement of AI systems to ensure their effectiveness, safety, and cost-efficiency over time; 5 Design AI solutions that are scalable and reproducible across different healthcare settings and patient populations; 6 Stay abreast of regulatory requirements; 7 Invest in recruiting and training healthcare professionals with AI, data science, and computational biology expertise to effectively leverage AI technologies in clinical practice.

By adopting these strategies, healthcare organizations can effectively manage costs while harnessing the transformative potential of AI to improve medical diagnosis, treatment, and patient outcomes.

This indicates that medical innovation is unsustainable unless we adopt new strategies and change historical trends. Bioscience advances will not be translated into patient benefits as they should. The potential of advances in genomics and other cutting-edge technologies will go unrealized or realized with long delays and huge investments lost. There are several gaps in translation at every stage, i.e., from discovery through development, regulatory approval, and reimbursement. This translates to many innovations in a journey of more than 20 years.

A significant mindset change from all the key stakeholders and actions is needed to implement sustainable healthcare, like integrated care across all health system levels. Patients and payers must be involved initially, not just at the end. Academicians must see themselves as part of an innovation process, not just as independent researchers. Regulators must be willing to accept more significant uncertainty. Companies and health systems should also focus on working together to ensure the proper treatment reaches the right patient at the right time rather than assuming a ‘free-for-all’ once marketing approval is granted.

AI has made many advances in healthcare efficiency. In particular, AI has improved the ability of physicians to diagnose diseases. According to the Institute of Medicine and National Academy of Science, engineering and medicine diagnostic errors contribute to approx. 10% of patient deaths. AI aims to mimic human cognitive functions and increase the availability of healthcare data.

The past few decades have seen considerable advances in diagnosing and treating cancer and other diseases. Yet, with the growing prevalence of cancer and other diseases and ongoing pressures on limited healthcare budgets, equal access to the latest scientific advances and their affordability have become a challenge. In the face of limited resources and increasing demand, we need to find better ways of allocating our resources and focus on what can make the most significant difference to patients. This results in eliminating interventions that offer limited benefit and prioritizing a patient stratification that gives the most critical benefit to patients, reducing inefficiency.

The need for AI in healthcare is clear. Healthcare relies on accuracy and intelligence with complex mechanisms of action and quality care needed to treat patients affected by different diseases.

As referenced in World Economic Forum, in June 2023 (“Emerging tech like AI is poised to make healthcare more accessible, accurate, and sustainable”, World Economic Forum), and by Pastarino et al. [127], one key element that will be essential for healthcare systems to pay attention to is whether adopting AI will make healthcare more sustainable.

This means that the technology should be designed with the purpose of longevity but also with the ability to adapt to a changing healthcare environment. In other words, a system designed for today's needs will be different from the needs of that same system in 20 years. This will require a consistent financial investment by institutions that should commit to ever-greening AI infrastructure for the future. There are a lot of hopes that AI will be able to advise the healthcare sector in various ways, making healthcare more sustainable and serving as an assistant to clinicians. This hope has been fueled by some successful applications of AI in healthcare. However, when we look at AI and healthcare side by side, there are unrealistic expectations of what AI can do and what the landscape of the healthcare industry will look like in the future.

## Regulatory considerations

As part of its digital strategy, the European Union (EU) seeks to regulate AI to create an environment for advancing and utilizing this groundbreaking technology. AI has the potential to yield a range of benefits, including enhanced healthcare, safer and more eco-friendly transportation, increased manufacturing efficiency, and more cost-effective, sustainable energy solutions. In April 2021, the European Commission introduced the initial regulatory framework for AI within the EU, which involves assessing and classifying AI systems that have the potential for various applications based on the level of risk they present to users. The extent of regulation will vary depending on the assessed risk levels. Once endorsed, these regulations will represent the world's pioneering rules concerning AI. The primary objective of the European Parliament is to ensure that AI systems utilized within the EU are safe, transparent, traceable, unbiased, and environmentally friendly. It is advocated that human oversight, as opposed to complete automation, be implemented to avert adverse consequences. Furthermore, the Parliament aims to create a consistent, technology-agnostic definition of AI that can be applied to future AI systems. The EU AI Draft proposes the first principles for Generative AI regulation; other countries will follow suit. The goal is to ensure that AI systems used in the EU are safe, transparent, traceable, non-discriminatory, and environmentally friendly (https://www.europarl.europa.eu/news/en/headlines/society/20230601STO93804/eu-ai-act-first-regulation-on-artificial-intelligence).

Generative AI, like ChatGPT, would have to comply with transparency requirements such as disclosing AI-generated content, designing the model to prevent it from generating illegal content, and publishing summaries of copyrighted data for training.

As reported recently, AI and ML technologies have significantly shaped many aspects of cancer care in recent years. Addressing the multiple sources of embedded bias to optimize these tools for cancer health equity requires a systemic, coordinated, and collaborative approach. Determinants of health and disease are multifactorial and complex, and our AI–ML technologies should reflect this complexity. Promoting health equity requires humanity, empathy, and transparency in data generation and AI–ML implementation efforts [128].

## Can AI facilitate faster and better healthcare?

The current debate about AI focuses on potential risks such as algorithmic bias and discrimination, loss of specific jobs, and other issues. Despite these dystopian scenarios, some scientists/physicians are focusing on the potential rewards of implementing AI. Some have argued that AI can solve some of the biggest and thorniest problems, drastically accelerating the pace of discovery in areas such as medicine, climate change, and green technology. Despite the challenges regarding the use of AI, two areas of research are promising. One is the so-called "literature-based discovery," which analyzes the scientific literature using ChatGPT-style language analysis to look for new hypotheses or hypotheses that might have been overlooked. This approach has already shown some promise, suggesting potential research collaborators among different stakeholders. This, in turn, will encourage interdisciplinary collaborations. The second area is "robot scientists" or "self-driving labs," which are robotic systems that use AI to form new hypotheses based on analysis of existing data and literature and then test those hypotheses by performing thousands of experiments in different fields, including systems biology. Unlike human scientists, robots are less driven by biases. Most of all, robots could scale up experimental research, develop unexpected theories, and explore avenues human investigators might have yet to consider. The idea that AI might transform scientific practice is feasible, though it will only happen for a while. One of the main barriers to the broad implementation of AI is that many scientists/physicians are concerned about possibly losing their jobs. Moreover, a recent report [129] showed that although Google Deepmind's AlphaFold showed in 2020 that it could predict the 3D structure of proteins with high accuracy, recent findings demonstrated that AlphaFold's prowess doesn’t yet translate into solid leads for drug-binding sites. Despite all these limitations, Al is now increasingly embraced by researchers in different fields.

According to a recent review by Michael Liebman [8], drug design and development provide a significant opportunity to apply AI-based methods and technologies. This has the potential to enhance success rates, expedite time to market, and reduce the costs associated with drug development [8].

Progress in drug development using AI has been limited, but there are firm hopes for the future. The technology needs to address specific critical challenges.

## Target selection

- How accurately is the disease/condition diagnosed and stratified, ensuring a well-defined phenotype?
- How comprehensive is patient stratification, considering clinical history, comorbidities, lifestyle, environmental factors, and genomics?
- Can the selected target be applied universally across the diverse real-world patient population, acknowledging observed diversity?

## Drug design/selection

- Can DL data be decoded effectively to interpret results?
- Considering comorbidities and polypharmacy in all patients, how are these factors considered?
- How effectively are pathway modulators being modeled concerning individual targets and responses?

## Clinical trials

- To what extent do inclusion and exclusion criteria reflect the characteristics of real-world patients?
- How does this impact the process of bringing a treatment to the market post-approval?
- Can the stratification of diseases and/or populations lead to more effective and efficient directed clinical trials?

These questions underscore the importance of addressing critical challenges in target selection, drug design/selection, and clinical trial design to harness the full potential of AI in drug discovery and development.

## AI: myth versus reality

There is a lot of hope that AI will advance the healthcare sector in various ways, not just for patient diagnosis, patient prognosis, and drug discovery, but also to assist physicians and provide better and more personalized treatment. This hope has been fueled by some successful applications of AI in healthcare. Side-by-side, however, there are unrealistic expectations of what AI can do and what the landscape of the healthcare industry will look like in the future.

Below, we listed two of the more common myths regarding the application of AI in healthcare:

1. AI will replace clinicians: While nobody can entirely predict the future, the fact is that physicians who understand the role of AI in healthcare likely have an advantage in their careers.
2. Programming knowledge is necessary to use AI successfully: AI in any field of study consists of many components, and programming is just one of them. Physicians and data scientists must continue collaborating to build meaningful AI systems for the continued growth, development, and success of AI applications in healthcare.

Like any other technology, AI comes with its advantages and disadvantages. AI biomarkers have several conceptual limitations:

1. Data quality is the first limitation of using AI. If we train a model with noise or artefactual images, many more cases will be necessary for the model to converge and achieve good performance [130]. To deploy AI models in medicine, large-scale validation studies with predefined performance metrics are required to guarantee model performance in the real world [131].
2. The training data must represent the real-world population [132]; if not, Deep Learning (DL) and Machine Learning (ML) models will fail. This is an issue in the medical context, where data distributions are markedly different between countries or hospitals. Without adequate precautions, such batch effects can inflate statistical results [132]. Mitigation strategies are to train on diverse datasets [133] or augment data [134].
3. AI models can be biased, meaning the performance depends on patient characteristics. To deploy AI models in medicine, large-scale validation studies with predefined performance metrics are required to guarantee model performance in the real world [131].
4. The fourth limitation is bias. AI models can be biased, meaning that the performance can depend on patient characteristics like age, gender, or ethnicity [70].
5. The fifth limitation is the quality of the ground truth. The model's performance will be limited to the established molecular biomarker predictive capacity when developing a model using molecular biomarkers as a surrogate of response to cancer immunotherapy.

We believe isolating the AI misconceptions and evaluating the future directions for AI in the medical field is essential. Not a day goes by with promising research studies on how to apply AI to the medical field. The term “artificial intelligence” itself might be misleading, given its meaning has been overinflated. An algorithm might perform very well on a pre-selected dataset. However, it should also be tested on real clinical data.

This issue in healthcare worldwide uses outdated infrastructure and technology Mi Ok Kim et al. [135] to address contemporary challenges and healthcare sustainability, which results in failure. Notably, the idea of sustainability for AI and healthcare is multifaceted and dramatically impacts the success of AI technologies. This is why, if not considered at the outset during its development, these challenges will fall to the management system within healthcare delivery, which has shown to be poorly equipped to respond to ongoing changes in technology and patient needs.

Moreover, as recently reported in the literature [5], the success of AI systems in real‐world applications depends on the capability of working accurately in a safe, reliable, and generalizable manner.

## AI: key limitations and outlook

It has been reported that increasing efforts to implement AI in PM to perform tasks such as disease diagnosis, predicting risks, and treatment responses are not without challenges. Indeed, many studies have shown promising experimental results (see Table 3). However, how AI improves health care still needs to be fully demonstrated. The limitations outlined for AI biomarkers highlight numerous challenges that must be addressed to deploy these models successfully in the medical field. Below, we describe a detailed assessment of the limitations and outlook of potential solutions:

The first limitation is data quality. Beyond the large amount of data that models require for achieving accurate, generalizable results, this data must be of high quality [130]. Suppose we train a model with noisy or artefactual images. In that case, many more cases will be necessary to converge for the model to perform well. The second limitation is generalization. DL models can fail to generalize if the training data does not represent real-world populations. This is especially an issue in a medical context, where data distributions vary markedly between countries or hospitals. Without adequate precautions, such batch effects can influence statistical performance [132]. There are mitigation strategies to train the training dataset on diverse datasets [133, 134].

The third limitation is bias. AI models can be biased, meaning that the performance can depend on patient characteristics like age, gender, or ethnicity [70]. To deploy AI models in medicine, large-scale validation studies with pre-defined performance metrics are required to guarantee model performance in the real world [131]. The fourth limitation is the quality of the ground truth. When developing a model using molecular biomarkers as a surrogate of response to immunotherapy, the model's performance will be limited to the established molecular biomarker predictive capacity.

## Data quality

- Challenge: High-quality data is crucial for training accurate and reliable AI models. Noisy or artefactual data can lead to biased or inaccurate predictions.
- Outlook: Emphasis should be placed on rigorous data curation and quality control. Initiatives to standardize data collection processes and ensure data integrity can improve the overall quality of the datasets used for training AI models.

## Generalization

- Challenge: Lack of representativeness in training data can result in poor generalization of AI models to diverse populations or real-world settings.
- Outlook: Strategies such as training on diverse datasets or augmenting data can help improve generalization. Collaborative efforts to pool data from different sources and regions may contribute to more robust and widely applicable models.

## Biases

- Challenge: AI models may exhibit biases based on patient characteristics, potentially leading to disparities in performance across different demographic groups.
- Outlook: Rigorous validation studies with diverse and well-characterized patient populations are essential. Ongoing efforts to develop and adhere to standardized guidelines for assessing and mitigating biases in AI models are critical. Transparency in reporting potential biases is crucial for building trust in AI applications.

## Quality of ground truth

- Challenge: The predictive capacity of AI models relying on molecular biomarkers is limited by the quality and reliability of the chosen biomarkers.
- Outlook: Continuous research and validation studies are necessary to establish and refine the predictive capacity of molecular biomarkers. Collaboration between researchers, clinicians, and data scientists can facilitate the identification of robust biomarkers and improve the overall quality of the ground truth used in AI model development.

In addition to these limitations, ongoing interdisciplinary collaboration, regulatory oversight, and ethical considerations are crucial for the responsible development and deployment of AI biomarkers in healthcare. As the field advances, addressing these limitations will be essential to ensure AI models' reliability, fairness, and generalizability in diverse medical settings.

To further optimize the integration of AI in drug development, clinical trials, and healthcare delivery, several future research directions and areas of innovation are recommended:

1. Deep Learning (DL) and Drug Discovery: Explore the application of DL techniques, such as deep neural networks and generative adversarial networks, in drug discovery. Research efforts can focus on virtual screening, de novo molecule design, and predicting drug-target interactions to accelerate the identification and optimization of novel therapeutic compounds.
2. Multi-Omics Data Integration: Investigate methods for integrating multi-omics data, including genomics, transcriptomics, proteomics, metabolomics, and microbiomics, to gain comprehensive insights into disease mechanisms and drug responses. Develop AI-driven approaches to analyze and interpret complex biological datasets, identify biomarkers, and personalize treatment strategies based on individual molecular profiles.
3. Clinical Trial Optimization with AI: Develop AI-driven algorithms for optimizing clinical trial design, patient recruitment, and endpoint selection. Explore methods for leveraging real-world evidence, electronic health records, wearable sensors, and mobile health technologies to streamline clinical trial operations, improve patient engagement, and enhance data quality and regulatory compliance.
4. Real-Time Monitoring and Predictive Analytics: Develop AI-driven systems for real-time monitoring, predictive analytics, and early warning systems in healthcare delivery settings. Research efforts can focus on developing algorithms for predicting patient deterioration, adverse events, and hospital readmissions, enabling proactive interventions and personalized care delivery.
5. Natural Language Processing (NLP) in Healthcare: Advance the application of natural language processing (NLP) techniques in healthcare for extracting insights from unstructured clinical notes, medical literature, and patient-generated content. Develop NLP models for clinical decision support, automated coding and documentation, and population health management, improving information retrieval and knowledge discovery in healthcare settings.
6. Federated Learning and Privacy-Preserving AI: Investigate federated learning and privacy-preserving AI techniques for collaborative model training and knowledge sharing across healthcare institutions while preserving patient privacy and data security. Develop secure and scalable frameworks for aggregating decentralized data sources, training robust models, and ensuring regulatory compliance in multi-institutional research collaborations.
7. Interoperability and Semantic Integration: Address interoperability challenges and semantic integration barriers in healthcare data systems to enable seamless exchange and integration of structured and unstructured data from diverse sources. Develop standards-based approaches for data representation, metadata management, and ontology mapping to facilitate data interoperability, semantic enrichment, and knowledge discovery across heterogeneous healthcare datasets.
8. Ethical, Legal, and Regulatory Frameworks: Develop ethical, legal, and regulatory frameworks for the responsible and transparent use of AI in drug development, clinical trials, and healthcare delivery. Address algorithmic bias, data privacy, informed consent, liability, and accountability concerns to ensure patient safety, equity, and trustworthiness in AI-enabled healthcare systems.

By prioritizing these research directions and fostering interdisciplinary collaborations between academia, industry, healthcare providers, and regulatory agencies, stakeholders can unlock the full potential of AI to transform drug development, clinical trials, and healthcare delivery, ultimately improving patient outcomes and advancing public healthcare.

## Conclusions

We are entering a new era characterized by an overflow of information but a need for more time and genuine human interaction. Technological advancements in AI algorithms equipped with PM have already resulted in an unprecedented acceleration of precision therapy, early disease detection, and personalized disease prevention strategies. The drug design and development field are poised to remain at the forefront of adopting emerging technologies. However, the critical question is whether these technologies should be integrated into the drug development process to improve existing pipelines and processes or restructure these processes considering these advancements. Overall, the synergy between AI and PM could ultimately decrease the disease burden for the public and, therefore, the cost, making preventive health care sustainable for all. However, we should remain vigilant in applying ethics and equity to ensure that these advancements do not increase health-related disparities, exacerbate existing inequities, or create new divides in care or health-related outcomes. The last pivotal element for successfully integrating AI and PM involves engaging all stakeholders, including researchers, healthcare professionals, citizens, and patients. This necessitates an investment in literacy, education, and capacity building to integrate AI into PM practices seamlessly.

## Review criteria

Publicly available information such as PubMed and the Internet were used for the literature review. We focused on identifying articles published on using AI and PM and sustainable health care. The research was restricted to the most recent studies in this field, and all research was limited to human studies published in English.

## Data Availability

All data supporting the findings of this study are available within the paper. The data presented in this study are openly available at https://pubmed.ncbi.nlm.nih.gov/, and https://clinicaltrials.gov/, all accessed in October 2023.

## Abbreviations

AI: Artificial intelligence
BM: Biomarkers
DL: Deep learning
DNN: Deep neural network
EHR: Electronic health records
IoMT: Internet of medical things
LLM: Large language model
ML: Machine learning
NLP: Natural language processing
PM: Precision medicine
SMILES: Simplified molecular input line entry system
